# Supplementary material for: How do household living conditions and gender-related decision-making influence child stunting in Rwanda? A population-based study
Source: PLoS One. 2024 Mar 13;19(3):e0290919. doi: 10.1371/journal.pone.0290919 (PMC10936808; doi:10.1371/journal.pone.0290919)
Supplement: S1 File — (PDF) [file pone.0290919.s001.pdf]

Software to use: STATASE 17

Step by step do file to recreate tables in the Manuscript

//Table 1: Sociodemographic characteristics

tab marital_status_n

tab a4_maritial_status

tab a14_education_level

tab education_mother_n

tab a14_education_level

tab EDUC_mother

tab educ_mother_01

tab years_schooling

tab education_mother_n

gen EDUC_MOTHER=1 if education_mother_n==0

replace EDUC_MOTHER=1 if education_mother_n==1

replace EDUC_MOTHER=2 if education_mother_n==2

replace EDUC_MOTHER=3 if education_mother_n==3

tab EDUC_MOTHER

label define EDUC_MOTHER 1"incomplete primary" 2"Complete primary" 3"secondary and above"

label values EDUC_MOTHER EDUC_MOTHER

tab EDUC_MOTHER

tab EDUC_MOTHER stunt, chi col

tab educ_partner_01

tab educ_husband

gen EDUC_FATHER=1 if educ_husband==1

replace EDUC_FATHER=1 if educ_husband==2

replace EDUC_FATHER=2 if educ_husband==3

```
replace EDUC_FATHER=3 if educ_husband==4

tab EDUC_FATHER

label define EDUC_FATHER 1"incomplete primary" 2"Complete primary" 3"secondary and above"

label values EDUC_FATHER EDUC_FATHER

tab EDUC_FATHER

tab EDUC_FATHER stunt, chi col

sum d8_birthweight

gen BW=1 if d8_birthweight<2500

replace BW=2 if d8_birthweight>2500& d8_birthweight<=2999

replace BW=3 if d8_birthweight>2999& d8_birthweight<=3499

replace BW=4 if d8_birthweight>3499& d8_birthweight<=3999

replace BW=5 if d8_birthweight>3999

tab BW

tab BW stunt, chi col

tab c11_number_anc_visits

tab c16_mode_of_delivery

tab c9_number_miscarriages

tab c4_children_stillborn

tab h1_today_overal_health

encode h1_today_overal_health, gen (H1)

tab H1

numlabel, add

tab H1

gen srh_mother=1 if H1==1

replace srh_mother=1 if H1==4

replace srh_mother=2 if H1==2

replace srh_mother=2 if H1==3

replace srh_mother=2 if H1==5

tab srh_mother
```

```
label define srh_mother 1"good" 2"poor"

label values srh_mother srh_mother

tab srh_mother

tab srh_mother stunt, chi col

tab H1

gen SRH=1 if H1==1

replace SRH=1 if H1==4

replace SRH=2 if H1==2

replace SRH=3 if H1==3

replace SRH=3 if H1==5

tab SRH

tab SRH stunt, chi col

tab a7_number_children_birth_to

gen parity=1 if a7_number_children_birth_to==1

replace parity=2 if a7_number_children_birth_to==2

replace parity=3 if a7_number_children_birth_to==3

replace parity=4 if a7_number_children_birth_to>=4

tab parity

tab parity stunt, chi col

gen PAR=1 if parity==1

replace PAR=1 if parity==2

replace PAR=2 if parity==3

replace PAR=2 if parity==4

replace PAR=3 if a7_number_children_birth_to>=5

tab PAR

tab PAR stunt, chi col

label define PAR 1" <=2 children" 2" 3-4 children" 3" >=5 children"

label values PAR PAR

tab PAR
```

tab a8_number_alive_children

tab c1_number_of_pregnancy

gen PREG=1 if c1_number_of_pregnancy==1

replace PREG=1 if c1_number_of_pregnancy==2

replace PREG=2 if c1_number_of_pregnancy==3

replace PREG=2 if c1_number_of_pregnancy==4

replace PREG=3 if c1_number_of_pregnancy>=5

tab PREG

tab PREG stunt, chi col

tab d45_diarrhea_in_last_2weeks

tab e19_still_breastfeeding

tab e19_still_breastfeeding stunt, chi col

///Partner's characteristics

We removed the 70 without partners at home.

tab A4_marital_range

preserve

drop if A4_marital_range==2

sum a20_partner_age

recode a20_partner_age (min/20=1) (21/34=2) (35/max=3), gen A20_partner_agerange

recode a20_partner_age (min/20=1) (21/34=2) (35/max=3), gen (A20_partner_agerange)

tab A20_partner_agerange

tab A20_partner_agerange stunt, chi col

tab a22_partner_education_level

encode a22_partner_education_level, gen (A22)

tab A22

numlabel, add

tab A22

tab A22

gen A22a=1 if A22==2

```
replace A22a=2 if A22==3
replace A22a=3 if A22==4
replace A22a=3 if A22==5
replace A22a=3 if A22==6
replace A22a=3 if A22==7
replace A22a=3 if A22==8
tab A22a
tab A22 stunt, chi col
tab A22a stunt, chi col
encode a26_partner_occupation, gen (A26)
tab A26
numlabel, add
tab A26
gen A26a=1 if A26==2
replace A26a=2 if A26==1
replace A26a=2 if A26==3
replace A26a=2 if A26==4
tab A26a
tab A26a stunt, chi col
//children characteristics
sum d8_birthweight, detail
ttest d8_birthweight, by(stunt)
ttest d_child_agemonths, by(stunt)
tab d45_diarrhea_in_last_2weeks
numlabel, add
encode d45_diarrhea_in_last_2weeks, gen(D45)
tab D45
gen diar=1 if D45==5
replace diar=1 if D45==4
```

```
replace diar=0 if D45==6
tab diar
label define diar 0"No" 1"Yes"
label values diar diar
tab diar
tab diar stunt, chi col
tab d54_illness_with_cough_last_2wee
tab D54
encode d54_illness_with_cough_last_2wee, gen(D54)
tab D54
numlabel, add
tab D54
gen cough=1 if D54==1
replace cough=1 if D54==2
replace cough=0 if D54==3
labeld define cough 0"No" 1"Yes"
label define cough 0"No" 1"Yes"
label values cough cough
tab cough
tab cough stunt, chi col
tab d52_fever_in_last_2weeks
encode d52_fever_in_last_2weeks, gen(D52)
tab D52
tab D52a
tab D52a stunt, chi col
tab d55_difficulty_breathing_last2we
encode d55_difficulty_breathing_last2we, gen(D55)
tab D55
numlabel, add
```

```
tab D55
gen breathe=0 if D55==1
replace breathe=0 if D55==2
replace breathe=1 if D55==3
tab breathe
label define breathe 0"No" 1"Yes"
label values breathe breathe
tab breathe
tab breathe stunt, chi col
tab HB
tab d38_is_child_ill_now
tab d38_is_child_ill_now stunt, chi col
ttest d9_haemoglobin, by(stunt)
tab d11_visible_severe_wasting
tab d11_visible_severe_wasting stunt, chi col
tab d13_child_overal_health
encode d13_child_overal_health, gen(D13)
tab D13
numlabel, add
tab D13
gen overall=0 if D13==2
replace overall=0 if D13==3
replace overall=0 if D13==5
replace overall=1 if D13==1
replace overall=1 if D13==4
label define overall 0"bad" 1"good"
label values overall overall
tab overall
tab overall overall
```

tab overall overall, chi col

tab overall stunt', chi col

tab overall stunt, chi col

///Table 2:"Living conditions and household assets"

tab a31_house_type

tab Roof

numlabel, add

tab Roof

encode Roof, gen(roof)

tab roof

numlabel, add

tab roof

gen a_roof=1 if roof==1

replace a_roof=1 if roof==2

replace a_roof=2 if roof==.

tab a_roof

drop a_roof

gen a_roof=1 if roof==1

replace a_roof=1 if roof==2

replace a_roof=0 if roof==.

tab a_roof

label define a_roof 0"No" 1"Yes"

label values a_roof a_roof

tab a_roof

tab a_roof stunt, chi col

tab Floor

```
encode Floor, gen(floor)
numlabel, add
tab floor
gen a_floor=0 if floor==2
replace a_floor=1 if floor==1
tab a_floor
tab a_floor stunt, chi col
tab Wall
encode Wall, gen(wall)
numlabel, add
tab wall
gen a_wall=0 if wall==5
replace a_wall=0 if wall==6
replace a_wall=1 if wall==1
replace a_wall=1 if wall==2
replace a_wall=1 if wall==3
replace a_wall=1 if wall==4
tab a_wall
tab a_wall stunt, chi col
tab a_floor
tab a_wall
tab a_roof
label define a_floor 0 "No" 1"Yes"
label values a_floor a_floor
label define a_wall 0"No" 1"Yes"
label values a_wall a_wall
tab a_wall
tab a_roof
tab a_floor
```

```
numlabel, add
tab a_floor
tab a_roof
tab a_wall
gen index_house=1 if a_floor==1&a_roof==1&a_wall==1
replace index_house=0 if a_floor==0&a_roof==0&a_wall==0
tab index_house
replace index_house=0 if a_floor==0&a_roof==1&a_wall==0
replace index_house=0 if a_floor==0&a_roof==1&a_wall==1
replace index_house=0 if a_floor==1&a_roof==0&a_wall==0
replace index_house=0 if a_floor==1&a_roof==1&a_wall==0
replace index_house=0 if a_floor==0&a_roof==0&a_wall==1
tab index
replace index_house=0 if a_floor==0&a_roof==1&a_wall==0
tab index
label define index_house 0"unimproved" 1"improved"
label values index_house index_house
tab index_house stunt, chi col
tab a32_drinking_water_source
encode a32_drinking_water_source, gen(A32)
numlabel, add
tab A32
gen water_source=1 if A32==2
replace water_source=1 if A32==4
replace water_source=0 if A32==1
tab water_source
label define water_source 0"improved" 1"unimproved"
label values water_source water_source
tab water_source stunt, chi col
```

```
tab a33_how_long_take_get_water
encode a33_how_long_take_get_water, gen(A33)
numlabel, add
tab A33
gen water_time=1 if A33==1
gen water_time=0 if A33==2&A33==3
replace water_time=0 if A33==2&A33==3
replace water_time=0 if A33==2|A33==3
tab water_time
label define water_time 0"No" 1"Yes"
label values water_time water_time
tab water_time
tab water_time stunt, chi col
gen index_water=0 if water_time==0&water_source==0
replace index_water=1 if water_time==1&water_source==0
replace index_water=1 if water_time==0&water_source==1
replace index_water=1 if water_time==1&water_source==1
tab index_water
label define index_water 0"improved" 1"unimproved"
label values index_water index_water
tab index_water stunt, chi col
encode a48_access_to_electricity, gen(A48)
numlabel, add
tab A48
gen electricity=0 if A48==2
replace electricity=1 if A48==1
tab elec
label define electricity 0 "Yes" 1"No"
label values electricity electricity
```

```
tab electricity

tab elec stunt, chi col

tab a38_kind_of_toilet

encode a38_kind_of_toilet, gen(A38)

numlabel, add

tab A38

gen toilet=0 of A38==1

gen toilet=0 if A38==1

replace toilet=1 if A38==2

tab toilet

label define toilet 0"Improved" 1"Unimproved"

label values toilet toilet

encode a39_share_toilet_with_other_fami, gen (A39)

numlabel, add

tab A39

gen shared_toilet=0 if A39==1

replace shared_toilet=1 if A39==2

label define shared_toilet 0"No" 1"Yes"

label values shared_toilet shared_toilet

tab shared_toilet shared_toilet

tab shared_toilet stunt, chi col

tab toilet stunt, chi col

gen improved_toilet=0 if toilet==0|shared_toilet==0

tab shared

numlabel, add

tab shared

tab toilet

replace improved_toilet=1 if toilet==0|shared_toilet==1

replace improved_toilet=1 if toilet==1|shared_toilet==1
```

```
replace improved_toilet=1 if toilet==1|shared_toilet==0
tab improved
drop improved
tab shared
tab toilet
gen TOILET=0 if toilet==0
replace TOILET=0 if shared_toilet==0
replace TOILET=1 if shared_toilet==0|toilet==1
replace TOILET=1 if shared_toilet==1|toilet==1
replace TOILET=1 if shared_toilet==1|toilet==0
tab TOILET
drop TOILET
gen TOILET=0 if toilet==0|shared_toilet==0
replace TOILET=1 if toilet==0|shared_toilet==1
replace TOILET=1 if toilet==1|shared_toilet==0
replace TOILET=1 if toilet==1|shared_toilet==1
tab TOI
gen TOILET=0 if toilet==0&shared_toilet==0
drop TOI
gen TOILET=0 if toilet==0&shared_toilet==0
replace TOILET=1 if toilet==0&shared_toilet==1
replace TOILET=1 if toilet==1&shared_toilet==0
replace TOILET=1 if toilet==1&shared_toilet==1
tab TOILET
label define TOILET 0"improved" 1"unimproved"
label values TOILET TOILET
tab TOILET stunt, chi col
tab a50_appliances
```

///Table 3: Calcualtion of the Multidimensional poverty index_asset

/Calculation of MPI

***3 dimensions: Health, Education , Living standards

//Health: Has 2 components: Nutrition and Child mortality

//Health: Nutrition

**Our outcome variable is undernutrition of children (1-36 months), in this dimension, we will ONLY use the BMI of mothers

**Undernutrition of the mothers by BMI<18.5

tab index_asset

drop index_asset

gen index_asset=0 if radio==0

tab index_asset

replace index_asset=0 if phone==1&television==1&moto==1&bicycle==1&car==1&refrigerator==1

tab index_asset

drop index_asset

gen index_asset=0 if radio==0&phone==1&television==1&moto==1&bicycle==1&car==1&refrigerator==1

tab index_asset

gen index_asset2=0 if phone==0&television==1&moto==1&bicycle==1&car==1&refrigerator==1&radio==1

tab index_asset2

gen index_asset3=0 if television==0&moto==1&bicycle==1&car==1&refrigerator==1&radio==1&phone==1

tab index_asset3

gen index_asset4=4 if &moto==0&bicycle==1&car==1&refrigerator==1&radio==1&phone==1&television==1

gen index_asset4=4 if moto==0&bicycle==1&car==1&refrigerator==1&radio==1&phone==1&television==1

gen index_asset4=0 if moto==0&bicycle==1&car==1&refrigerator==1&radio==1&phone==1&television==1

drop index_asset4

```
gen index_asset4=0 if
moto==0&bicycle==1&car==1&refrigerator==1&radio==1&phone==1&television==1

tab index_asset4

gen index_asset5=0 if
car==0&bicycle==1&moto==1&refrigerator==1&radio==1&phone==1&television==1

tab a49_bic_mot_car

tab a49_bic_mot_car a50_appliances

tab A49

tab A50

gen asset_index=0 if A50==3|A50==4|A50==5|A50==6|A50==9|A50==10|A50==12|A50==17|A50==18

replace asset_index=1 if
A50==1|A50==2|A50==11|A50==8|A50==7|A50==13|A50==14|A50==15|A50==16

tab asset_index

tab A49

gen asset_indexB=0 if A49==2

replace asset_indexB=0 if A49==1|A49==3|A49==4|A49==5|A49==6|A49==7

tab asset_indexB

drop asset_indexB

gen asset_indexB=0 if A49==2

replace asset_indexB=1 if A49==1|A49==3|A49==4|A49==5|A49==6|A49==7

tab asset_indexB

gen composite_asset=0 if asset_index==0|asset_indexB==0

replace composite_asset=1 if asset_index==1|asset_indexB==1

tab composite

tab asset_index stunt, chi col

save "C:\Users\xutuje\OneDrive - University of Gothenburg\Dr U_PhD file\Datasets\09March2023
Dataset_current.dta", replace

tab asset_index

label define asset_index 0"Less than 2" 1"more than 2"

label values asset_index asset_index
```

```
tab asset_index stunt, chi col

tab education_mother

tab d9_weightofmother

tab BMI_mother

recode BMI_mother (min/18.49999=0"underweight" 18.5/max=1"Normal"), gen(mother_nutrition)

recode BMI_mother (min/18.49999=0"underweight") ( 18.5/max=1"Normal"), gen(mother_nutrition)

recode BMI_mother(min/18.49999=0"underweight")(18.5/max=1"Normal"), gen(mother_nutrition)

recode BMI_mother(min/18.49999=0 "underweight")(18.5/max=1"Normal"), gen(mother_nutrition)

recode BMI_mother(min/18.49999=0 "underweight")(18.5/max=1 "Normal"), gen(mother_nutrition)

tab mother_nutrition

tab education_mother

tab education_partner

gen educ_mpi=0 if education_mother==0& education_partner==0

replace educ_mpi=1 if education_mother==1& education_partner==1

replace educ_mpi=1 if education_mother==2& education_partner==2

tab educ_mpi

replace educ_mpi=1 if education_mother==0& education_partner==1

replace educ_mpi=1 if education_mother==0& education_partner==2

replace educ_mpi=1 if education_mother==1& education_partner==0

replace educ_mpi=1 if education_mother==2& education_partner==0

replace educ_mpi=1 if education_mother==1& education_partner==2

replace educ_mpi=1 if education_mother==2& education_partner==2

replace educ_mpi=1 if education_mother==2& education_partner==1

tab educ_mpi

label define educ_mpi 0"<6years_educ" 1">6 years_educ"

label values educ_mpi educ_mpi

tab a15_total_years_school_and_unive

tab educ_mpi stunt, chi col

encode a15_total_years_school_and_unive (mi/5.999=0 "less than 6") (6/max=1 "More than 6")
```

```
encode a15_total_years_school_and_unive (min/5.999=0 "less than 6") (6/max=1 "More than 6"), gen (mother_schools)

recode a15_total_years_school_and_unive (min/5.999=0 "less than 6") (6/max=1 "More than 6"), gen (mother_schools)

tab mother_sc

recode a23_paertner_total_years_school_ (min/5.999=0 "less than 6") (6/max=1 "More than 6"), gen (partner_schools)

tab partner_sc

gen educ_mpiB=0 if mother_schools ==0& partner_schools ==0

replace educ_mpiB=1 if mother_schools ==1& partner_schools ==0

replace educ_mpiB=1 if mother_schools ==1& partner_schools ==1

replace educ_mpiB=1 if mother_schools ==0& partner_schools ==1

tab educ_mpiB

educ mpi_B stunt, chi col

tab educ_mpiB stunt, chi col

tab a23_paertner_total_years_school_

tab educ_mpiB A4_marital_status

tab A4_marital_status

replace educ_mpiB=1 if mother_schools ==0& partner_schools ==1

replace educ_mpiB=1 if mother_schools ==1

tab educ_mpiB

tab educ_mpiB A4_marital_status

replace educ_mpiB=0 if mother_schools ==0

tab educ_mpiB

tab educ_mpiB A4_marital_status

tab educ_mpiB stunt, chi col

mpi d1( mother_nutrition ) d2( educ_mpiB ) d3( TOILET index_water electricity index_house asset_index ), cutoff(0.3)

tab index water

tab index_water
```

```
tab index_asset
tab asset_index stunt, chi col
numlabel add
numlabel, add
tab asset_index
gen asset_indexB=0 if asset_index==1
gen assetIndex=0 if asset_index==1
replace assetIndex=1 if asset_index==0
tab assetIndex
tab educ_mpiB
gen Educmpi=0 if educ_mpiB==1
replace Educmpi=1 if educ_mpiB==0
tab Educmpi
tab mother_nutrition
gen BMImpi=0 if mother_nutrition==1
replace BMImpi=1 if mother_nutrition==0
tab BMImpi
tab TOILET
tab electricity
index_house
tab index_house
gen housempi=0 if index_house==1
replace housempi=1 if index_house==0
tab housempi
tab index_water
tab assetIndex
mpi d1( BMImpi ) d2( Educmpi ) d3( TOILET index_water electricity housempi asset_index ), cutoff(0.3)
rename TOILET toilet_mpi
rename electicity electicity_mpi
```

```
rename electricity electricity_mpi

index_water

tab index_water

rename index_water water_mpi

rename asset_index asset_mpi

tab asset_mpi

tab assetIndex

mpi d1( BMImpi ) d2( Educmpi ) d3( TOILET index_water electricity housempi assetIndex ), cutoff(0.3)

mpi d1( BMImpi ) d2( Educmpi ) d3( toilet_mpi water_mpi electricity_mpi housempi assetIndex ),
cutoff(0.3)

tab housempi

tab Educmpi

labed define housempi 0"improved" 1"unimproved"

label define housempi 0"improved" 1"unimproved"

label values housempi housempi

numlabel, add

tab housempi

label define BMImpi 0"improved" 1"unimproved"

label values BMImpi BMImpi

tab BMImpi

numlabel, add

tab BMImpi

tab housempi

tab Educmpi

label define Educmpi 0"improved" 1"unimproved"

label values Educmpi Educmpi

tab Educmpi

numlabel, add

tab Educmpi
```

tab asset_mpi

tab assetIndex

label define assetIndex 0"improved" 1"unimproved"

label values assetIndex assetIndex

tab assetIndex

numlabel , add

tab assetIndex

rename assetIndex asset_mpi

drop asset_mpi

rename assetIndex asset_mpi

rename educ_mpiB education_combined

mpi d1(BMImpi) d2(Educmpi) d3(toilet_mpi water_mpi electricity_mpi housempi asset_mpi), cutoff(0.3)

mpi d1(BMImpi) d2(Educmpi) d3(toilet_mpi water_mpi electricity_mpi housempi asset_mpi), cutoff(0.3)

///Table 4: Social support and Child stunting

>>>Social support and Child stunting

Making the 2 groups: Stunted and Normal kids

generate stunt=1 if HAZRec==1

replace stunt=1 if HAZRec==2

replace stunt=0 if HAZRec==3

>> Defining the 2 groups

label define stunt 0"Normal" 1"Stunted"

label values stunt stunt

>>Table about social support (from b1 to b6 questions)

>>Social support during illness

```
tab b1_have_friend_to_assist_when_il
encode b1_have_friend_to_assist_when_il, gen(B1ill)
tab B1ill
numlabel, add
tab B1ill
gen b1ill=0 if B1ill==2
replace b1ill=1 if B1ill==1
replace b1ill=1 if B1ill==3
replace b1ill=1 if B1ill==4
tab b1ill
label define b1ill 0"No" 1"Yes"
label values b1ill b1ill
tab b1ill
>>> Social support about food
tab b2_share_food_with_you
encode b2_share_food_with_you, gen(B2food)
tab B2food
numlabel, add
tab B2food
gen b2food=0 if B2food==2
replace b2food=1 if B2food==1
replace b2food=1 if B2food==3
replace b2food=1 if B2food==4
tab b2food
label define b2food 0"No" 1"Yes"
label values b2food b2food
tab b2food
>>> Social support about house
tab b3_share_their_house_with_you
```

```
encode b3_share_their_house_with_you, gen(B3house)

tab B3house

numlabel, add

tab B3house

gen b3house=0 if B3house==2

replace b3house=1 if B3house==1

replace b3house=1 if B3house==3

replace b3house=1 if B3house==4

tab b3house

label define b3house 0"No" 1"Yes"

label values b3house b3house

tab b3house

>>>Social support about money

tab b4_lend_you_money

encode b4_lend_you_money, gen(B4money)

tab B4money

numlabel, add

tab B4money

gen b4money=0 if B4money==2

replace b4money=1 if B4money==1

replace b4money=1 if B4money==3

replace b4money=1 if B4money==4

tab b4money

label define b4money 0"No" 1"Yes"

label values b4money b4money

tab b4money

>>>Social support during problems

tab b5_help_guidance_in_problems

encode b5_help_guidance_in_problems, gen(B5problems)
```

tab B5problems

numlabel, add

tab B5problems

gen b5problems=0 if B5problems==2

replace b5problems=1 if B5problems==1

replace b5problems=1 if B5problems==3

replace b5problems=1 if B5problems==4

tab b5problems

label define b5problems 0"No" 1"Yes"

label values b5problems b5problems

tab b5problems

>>>Social support during personal problems

tab b6_support_in_personal_problems

encode b6_support_in_personal_problems, gen(B6personal)

tab B6personal

numlabel, add

tab B6personal

gen b6personal=0 if B6personal==2

replace b6personal=1 if B6personal==1

replace b6personal=1 if B6personal==3

replace b6personal=1 if B6personal==4

tab b6personal

label define b6personal 0"No" 1"Yes"

label values b6personal b6personal

tab b6personal

>>>Calculation of Crude Odds Ratio (COR)

logistic stunt ib1.b1ill

logistic stunt ib1.b2food

logistic stunt ib1.b3house

logistic stunt ib1.b4money

logistic stunt ib1.b5problems

logistic stunt ib1.b6personal

✓Calculation of adjusted Odd Ratio (AOR)

✓✓"Mother's age-range"

recode a1_age (min/20=1 ">=20") (21/34=2 "21-34") (35/max=3 ">=35"), gen(a1_agerange)

✓✓ "Partner's age-range"

recode a20_partner_age (min/20=1 ">=20") (21/34=2 "21-34") (35/max=3 ">=35"), gen(A20_agerange)

✓✓ "Mother's education level"

encode a14_education_level, gen(A14)

gen education_mother=0 if A14==1

replace education_mother=0 if A14==2

replace education_mother=1 if A14==3

replace education_mother=2 if A14==4

replace education_mother=2 if A14==5

replace education_mother=2 if A14==6

replace education_mother=2 if A14==7

replace education_mother=2 if A14==8

replace education_mother=2 if A14==9

tab education_mother

tab education_mother stunt, chi col

✓✓"Partner education level"

```
encode a22_partner_education_level, gen(A22)
gen education_partner=0 if A22==1
replace education_partner =0 if A22==2
replace education_partner =1 if A22==3
replace education_partner =2 if A22==4
replace education_partner =2 if A22==5
replace education_partner =2 if A22==6
replace education_partner =2 if A22==7
replace education_partner =2 if A22==8
tab A22
tab education_partner
```

>>"Marital status must be removed from Partner's data"

```
tab a4_maritial_status
encode a4_maritial_status, gen (marital_status)
numlabel, add
tab marital_status
gen A4_marital_status=1 if marital_status==1
replace A4_marital_status=1 if marital_status==3
replace A4_marital_status=1 if marital_status==4
replace A4_marital_status=2 if marital_status==2
replace A4_marital_status=2 if marital_status==5
replace A4_marital_status=2 if marital_status==6
tab A4
preserve
drop if A4_marital_status==2
```

```
tab A22

tab education_partner

tab education_partner stunt, chi col

restore


>>"Household income"

tab a27_total_household_income

encode a27_total_household_income, gen(A27_income)

numlabel , add

tab A27

gen income=1 if A27==6

replace income=2 if A27==2

replace income=3 if A27==1

replace income=3 if A27==3

replace income=3 if A27==4

replace income=3 if A27==7

tab income

tab income stunt, chi col

>>"sex of the head of the household"


tab a10_sex_of_household_head

encode a10_sex_of_household_head, gen (A10_sex_hh)

tab A10_sex_hh stunt, chi col


>>>>"Adjusted Odd ratio"

logistic stunt ib1.b1ill i.a1_agerange i.A20_agerange i.education_mother i.education_partner i.A10_sex_hh i.income

logistic stunt ib1.b2food i.a1_agerange i.A20_agerange i.education_mother i.education_partner i.A10_sex_hh i.income
```

logistic stunt ib1.b3house i.a1_agerange i.A20_agerange i.education_mother i.education_partner i.A10_sex_hh i.income

logistic stunt ib1.b4money i.a1_agerange i.A20_agerange i.education_mother i.education_partner i.A10_sex_hh i.income

logistic stunt ib1.b5problems i.a1_agerange i.A20_agerange i.education_mother i.education_partner i.A10_sex_hh i.income

logistic stunt ib1.b6personal i.a1_agerange i.A20_agerange i.education_mother i.education_partner i.A10_sex_hh i.income

////Table 5: Household decision-making and child stunting

/Table 5: Decision making of women and association with child stunting: DO file for the revised COR and AOR

use "C:\Users\xutuje\OneDrive - University of Gothenburg\Dr U_PhD file\Datasets\PhD DATASET AUGUST_COMPOSITE.dta"

logistic stunt i.electricity_mpi i2.educ_p i2.educ_2 i2.incomeviolence i2.occup_partner i2.occup_women

logistic stunt i.housempi i2.educ_p i2.educ_2 i2.incomeviolence i2.occup_partner i2.occup_women

logistic stunt i.water_source i2.educ_p i2.educ_2 i2.incomeviolence i2.occup_partner i2.occup_women

logistic stunt i.water_time i2.educ_p i2.educ_2 i2.incomeviolence i2.occup_partner i2.occup_women

logistic stunt i1.water_time i2.educ_p i2.educ_2 i2.incomeviolence i2.occup_partner i2.occup_women

logistic stunt i0.water_time i2.educ_p i2.educ_2 i2.incomeviolence i2.occup_partner i2.occup_women

logistic stunt i0.toilet i2.educ_p i2.educ_2 i2.incomeviolence i2.occup_partner i2.occup_women

logistic stunt i0.toilet_mpi i2.educ_p i2.educ_2 i2.incomeviolence i2.occup_partner i2.occup_women

logistic stunt i1.toilet_mpi i2.educ_p i2.educ_2 i2.incomeviolence i2.occup_partner i2.occup_women

tab radio

logistic stunt i.radio i2.educ_p i2.educ_2 i2.incomeviolence i2.occup_partner i2.occup_women

logistic stunt i.phone i2.educ_p i2.educ_2 i2.incomeviolence i2.occup_partner i2.occup_women

logistic stunt i.television i2.educ_p i2.educ_2 i2.incomeviolence i2.occup_partner i2.occup_women

logistic stunt i.bicycle i2.educ_p i2.educ_2 i2.incomeviolence i2.occup_partner i2.occup_women

logistic stunt i.asset_mpi i2.educ_p i2.educ_2 i2.incomeviolence i2.occup_partner i2.occup_women

```
logistic stunt i.composite_asset i2.educ_p i2.educ_2 i2.incomeviolence i2.occup_partner i2.occup_women

logistic stunt i0.composite_asset i2.educ_p i2.educ_2 i2.incomeviolence i2.occup_partner i2.occup_women

tab index_asset

tab composite_asset

tab asset_mpi

logistic stunt i.asset_mpi

logistic stunt i0.asset_mpi

logistic stunt i.10 asset_mpi i2.educ_p i2.educ_2 i2.incomeviolence i2.occup_partner i2.occup_women

logistic stunt i0. asset_mpi i2.educ_p i2.educ_2 i2.incomeviolence i2.occup_partner i2.occup_women

logistic stunt i.bicycle i2.educ_p i2.educ_2 i2.incomeviolence i2.occup_partner i2.occup_women

tab purchases

logistic stunt i.purchases

logistic stunt i2.purchases

logistic stunt i4.purchases

tab purchases

tab i1_decision_major_purchases

tab purchases

tab health

gen dec-purchase=1 if purchases==1

gen decPurchase=1 if purchases==1

replace decPurchase=1 if purchases==3

tab purchases

tab purchases

gen A=1 if purchases==1

gen A=1 if purchases==1

replace A=1 if purchases==2

replace A=1 if purchases==3
```

```
replace A=2 if purchases==4

replace A=3 if purchases==5

tab A

label define A 1"Only Partner/others in family" 2"Personal decision" 3"Joint decision with partner"

label values A A

tab A

logistic stunt i2.A

tab A stunt, chi col

logistic stunt i2.A

logistic stunt i.A

logistic stunt i1.A

logistic stunt i3.A

tab A

numlabel, add

tab A

logistic stunt i.A i2.educ_p i2.educ_2 i2.incomeviolence i2.occup_partner i2.occup_women

logistic stunt i1.A

logistic stunt i1.A i2.educ_p i2.educ_2 i2.incomeviolence i2.occup_partner i2.occup_women

tab family_visit

gen B=1 if family_visit==5

replace B=2 if family_visit==4

replace B=3 if family_visit==1

replace B=3 if family_visit==3

replace B=3 if family_visit==2

tab B

logistic stunt i.B

label define B 1"Joint!" 2"Personal"  3"others"

label values B B

tab B
```

```
logistic stunt i1.A i2.educ_p i2.educ_2 i2.incomeviolence i2.occup_partner i2.occup_women
logistic stunt i.B
logistic stunt i.B i2.educ_p i2.educ_2 i2.incomeviolence i2.occup_partner i2.occup_women
tab money
gen C=1 if money==5
replace C=2 if money==4
replace C=3 if money==1
replace C=3 if money==2
replace C=3 if money==3
tab C
label define C 1"Joint" 2"Personal" 3"Others"
label values C C
tab C
logistic stunt i.C
logistic stunt i.C i2.educ_p i2.educ_2 i2.incomeviolence i2.occup_partner i2.occup_women
tab health
gen D=1 if health==5
replace D=2 if health==4
replace D=3 if health==1
replace D=3 if health==2
replace D=3 if health==3
tab D
label define D 1"Joint" 2"Personal" 3"Others"
label values D D
tab D
logistic stunt i.D
logistic stunt i.D i2.educ_p i2.educ_2 i2.incomeviolence i2.occup_partner i2.occup_women
tab FP_decision
gen D=1 if FP_decision==7
```

```
tab D
gen E=1 if FP_decision==7
replace E=2 if FP_decision==6
replace E=3 if FP_decision==1
replace E=3 if FP_decision==2
replace E=3 if FP_decision==3
tab E
label define E 1"Joint" 2"Personal" 3"Others"
label values E E
tab E
logistic stunt i.E
logistic stunt i.E i2.educ_p i2.educ_2 i2.incomeviolence i2.occup_partner i2.occup_women
tab i8_can_say_no_to_sexual_intercou
tab I8
encode i8, gen I8
encode i8, gen (I8)
tab I8
numlabel.
numlabel, add
tab I8'
tab I8
gen F=1 if I8==2
replace F=2 if I8==1
replace F=2 if I8==3
tab F
label define F 1"No" 2 "Yes"
label values F F
tab F
logistic stunt i.F
```

```
logistic stunt i2.F
logistic stunt i1.F
logistic stunt i1.F i2.educ_p i2.educ_2 i2.incomeviolence i2.occup_partner i2.occup_women
tab i9_can_ask_use_candom
encode i9_can_ask_use_candom, gen (I9)
tab I9
numlabel, add
tab I9
gen condom=1 if I9==3
replace condom=2 if I9==1
replace condom=2 if I9==2
tab condom
label define condom 1"Yes" 2"No"
label values condom condom
tab condom
logistic stunt i1.condom
logistic stunt i2.condom
logistic stunt i2.condom i2.educ_p i2.educ_2 i2.incomeviolence i2.occup_partner i2.occup_women
tab land
gen G=1 if land==2
replace G=2 if land==3
replace G=3 if land==1
label define G 1"Joint" 2"personal" 3"None"
label values G G
tab G
logistic stunt i.G
logistic stunt i2.G i2.educ_p i2.educ_2 i2.incomeviolence i2.occup_partner i2.occup_women
logistic stunt i.G i2.educ_p i2.educ_2 i2.incomeviolence i2.occup_partner i2.occup_women
tab k1_land_ownership
```

```
tab i4_own_house

encode i4_own_house, gen (I4)

tab I4

numlabel , add

tab I4

gen H=1 if I4==2

replace H=2 if I4==3

replace H=3 if I4==1

label define H 1"Joint" 2"Personal" 3"None"

label values H H

tab H

logistic stunt i.H

logistic stunt i.H i2.educ_p i2.educ_2 i2.incomeviole
```

END
